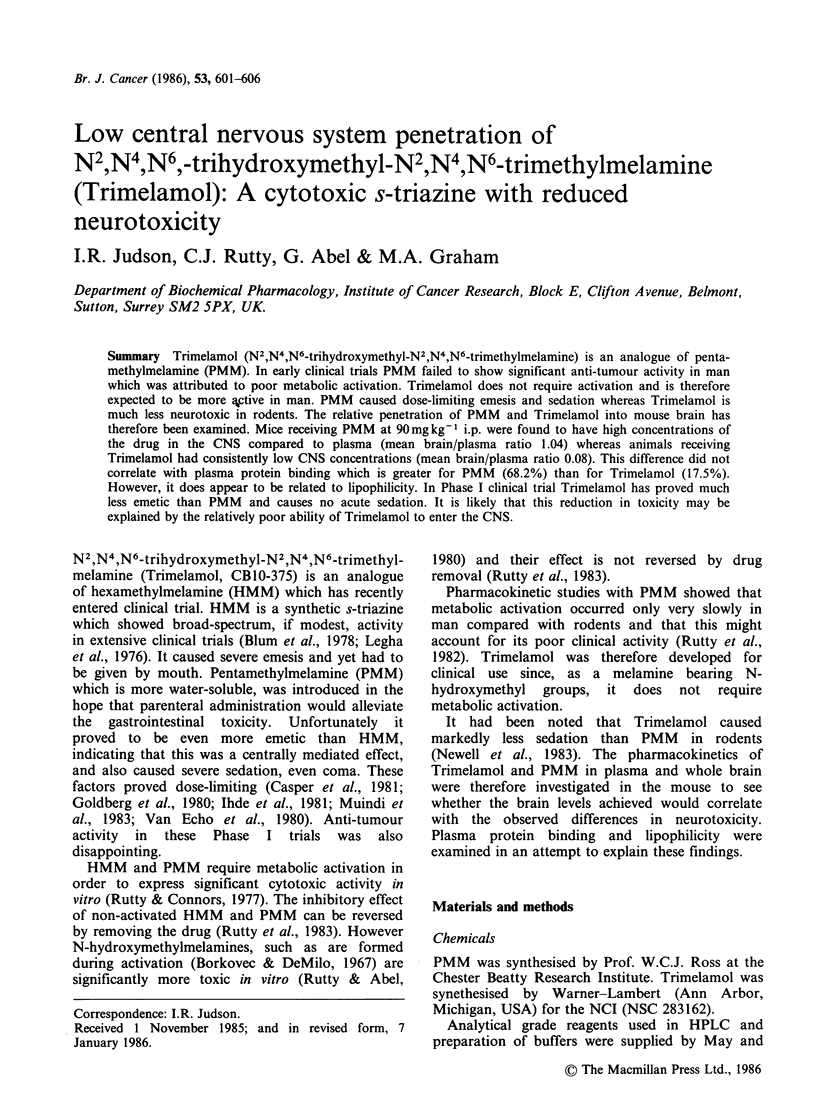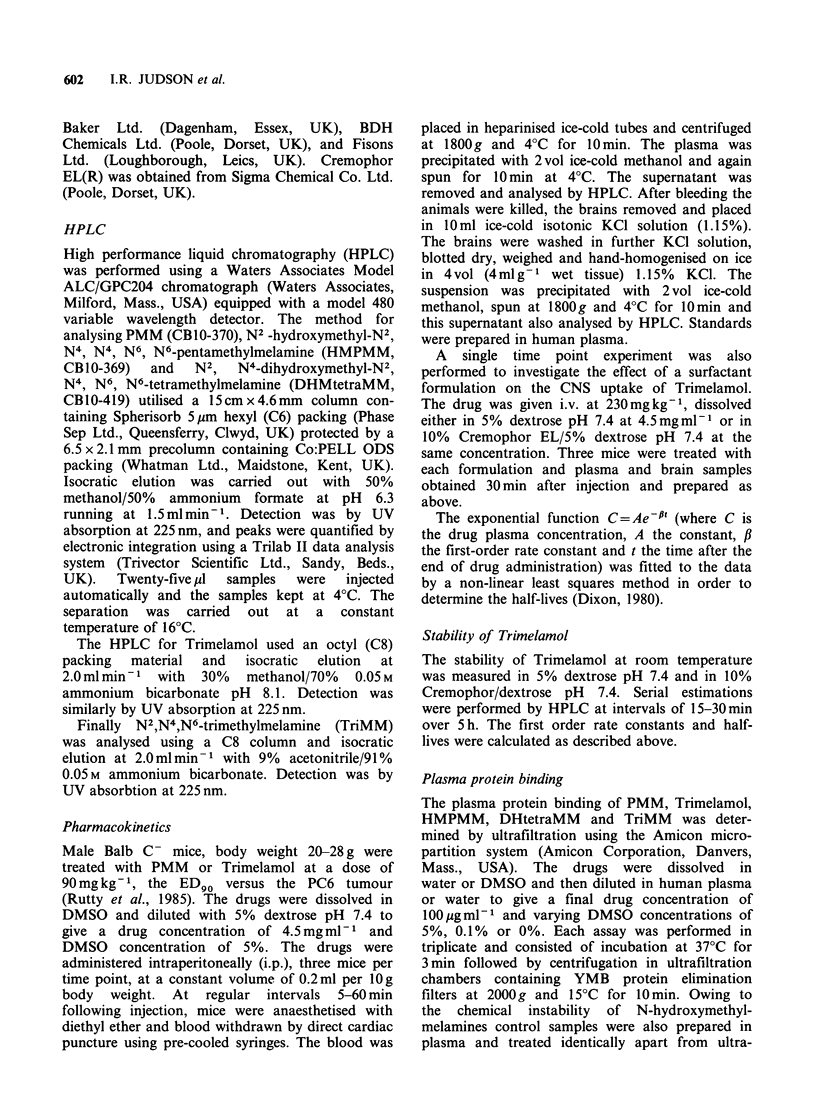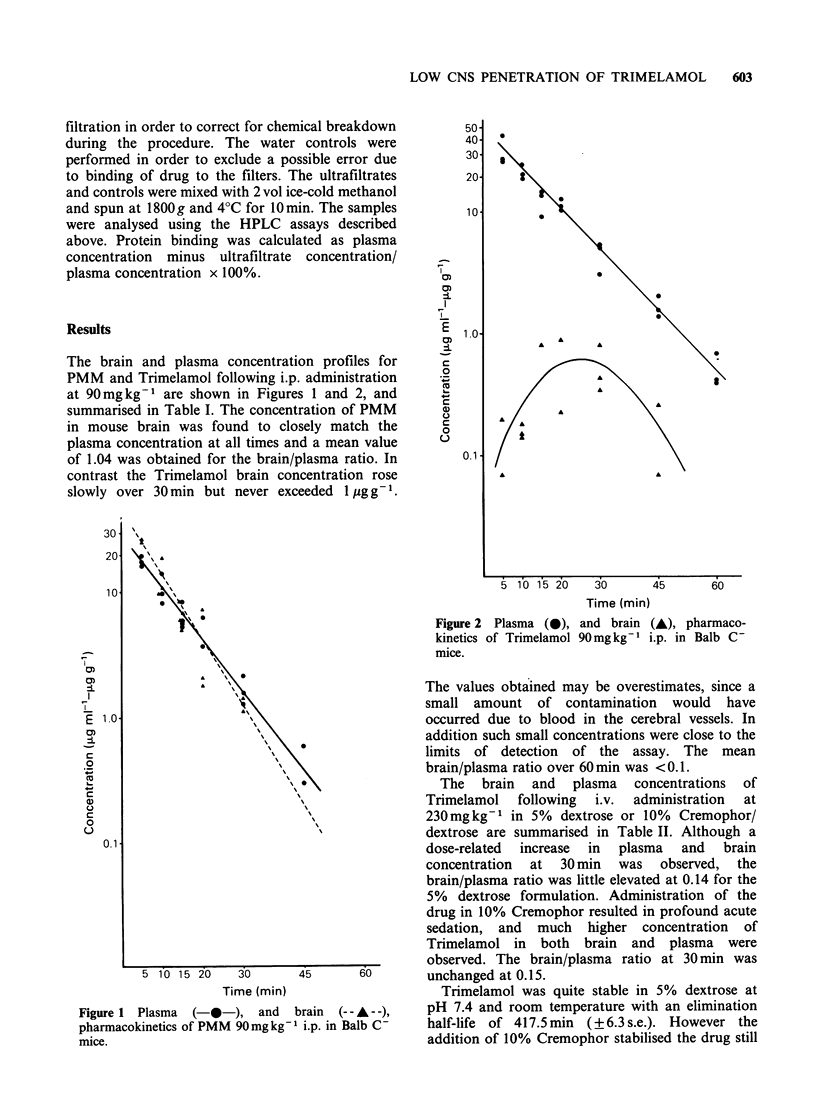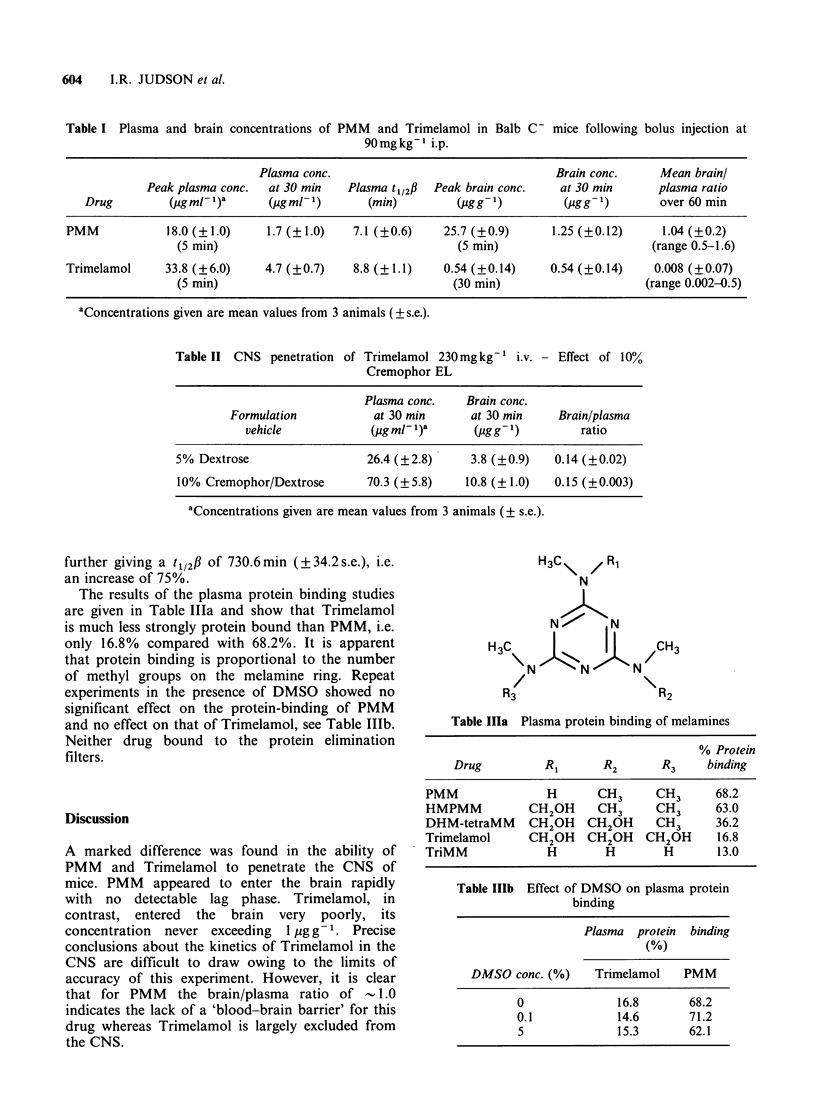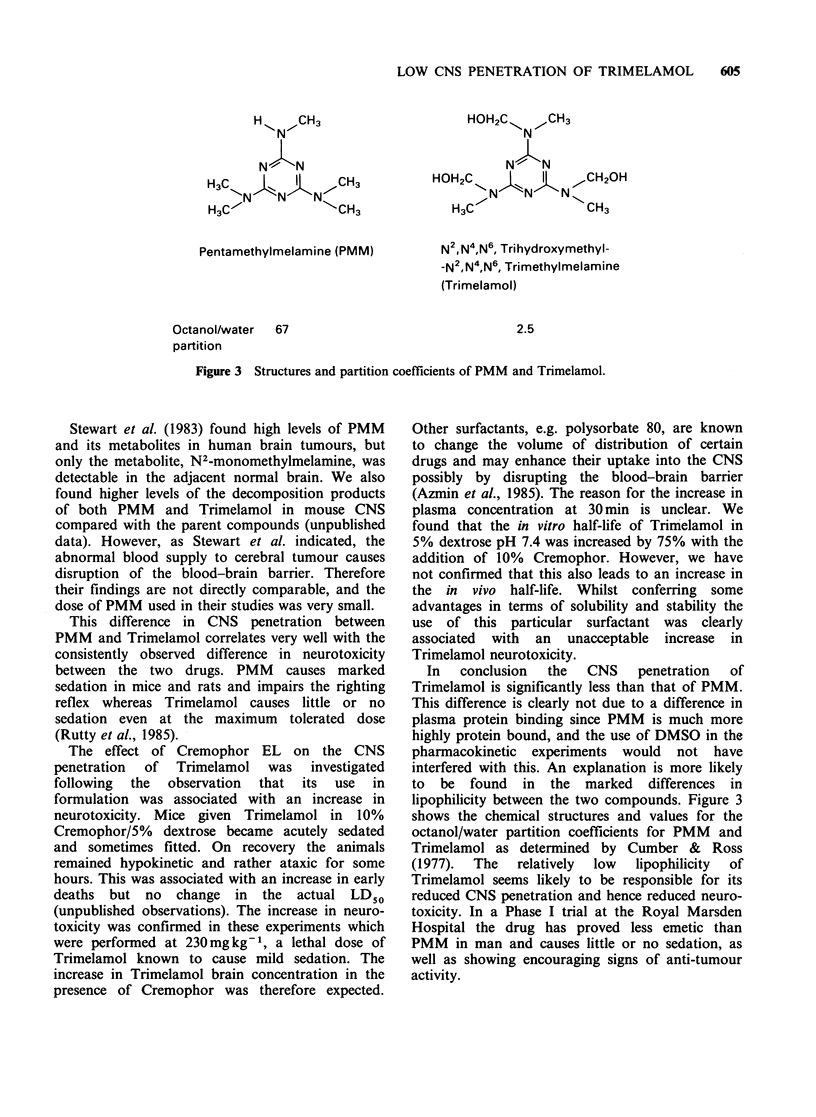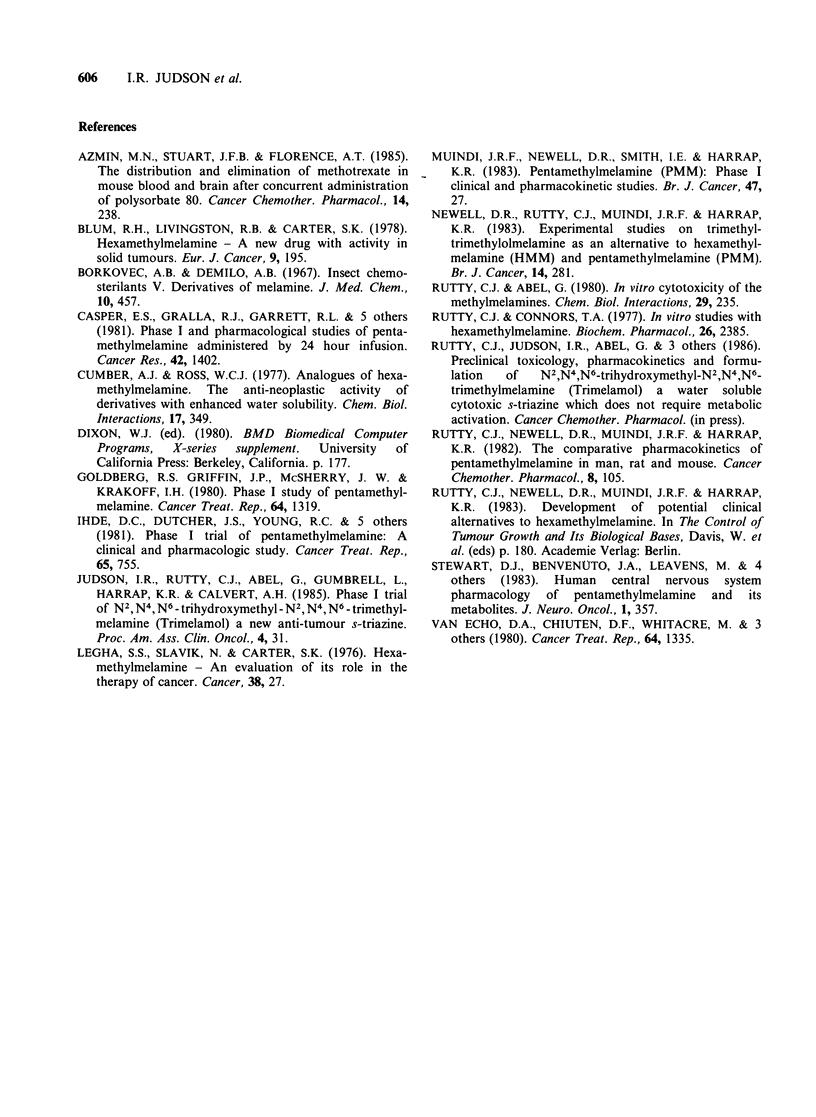# Low central nervous system penetration of N2,N4,N6,-trihydroxymethyl-N2,N4,N6,-trimethylmelamine (Trimelamol): a cytotoxic s-triazine with reduced neurotoxicity.

**DOI:** 10.1038/bjc.1986.102

**Published:** 1986-05

**Authors:** I. R. Judson, C. J. Rutty, G. Abel, M. A. Graham

## Abstract

Trimelamol (N2,N4,N6-trihydroxymethyl-N2,N4,N6-trimethylmelamine) is an analogue of pentamethylmelamine (PMM). In early clinical trials PMM failed to show significant anti-tumour activity in man which was attributed to poor metabolic activation. Trimelamol does not require activation and is therefore expected to be more active in man. PMM caused dose-limiting emesis and sedation whereas Trimelamol is much less neurotoxic in rodents. The relative penetration of PMM and Trimelamol into mouse brain has therefore been examined. Mice receiving PMM at 90 mg kg-1 i.p. were found to have high concentrations of the drug in the CNS compared to plasma (mean brain/plasma ratio 1.04) whereas animals receiving Trimelamol had consistently low CNS concentrations (mean brain/plasma ratio 0.08). This difference did not correlate with plasma protein binding which is greater for PMM (68.2%) than for Trimelamol (17.5%). However, it does appear to be related to lipophilicity. In Phase I clinical trial Trimelamol has proved much less emetic than PMM and causes no acute sedation. It is likely that this reduction in toxicity may be explained by the relatively poor ability of Trimelamol to enter the CNS.


*Br. J. Cancer* (1986), **53**, 601–606

### Low central nervous system penetration of $N^2,N^4,N^6$,-trihydroxymethyl-$N^2,N^4,N^6$-trimethylmelamine (Trimelamol): A cytotoxic *s*-triazine with reduced neurotoxicity

I.R. Judson, C.J. Rutty, G. Abel & M.A. Graham

*Department of Biochemical Pharmacology, Institute of Cancer Research, Block E, Clifton Avenue, Belmont, Sutton, Surrey SM2 5PX, UK.*

**Summary** Trimelamol ($N^2,N^4,N^6$-trihydroxymethyl-$N^2,N^4,N^6$-trimethylmelamine) is an analogue of pentamethylmelamine (PMM). In early clinical trials PMM failed to show significant anti-tumour activity in man which was attributed to poor metabolic activation. Trimelamol does not require activation and is therefore expected to be more active in man. PMM caused dose-limiting emesis and sedation whereas Trimelamol is much less neurotoxic in rodents. The relative penetration of PMM and Trimelamol into mouse brain has therefore been examined. Mice receiving PMM at $90\,mg\,kg^{-1}$ i.p. were found to have high concentrations of the drug in the CNS compared to plasma (mean brain/plasma ratio 1.04) whereas animals receiving Trimelamol had consistently low CNS concentrations (mean brain/plasma ratio 0.08). This difference did not correlate with plasma protein binding which is greater for PMM (68.2%) than for Trimelamol (17.5%). However, it does appear to be related to lipophilicity. In Phase I clinical trial Trimelamol has proved much less emetic than PMM and causes no acute sedation. It is likely that this reduction in toxicity may be explained by the relatively poor ability of Trimelamol to enter the CNS.

$N^2,N^4,N^6$-trihydroxymethyl-$N^2,N^4,N^6$-trimethylmelamine (Trimelamol, CB10-375) is an analogue of hexamethylmelamine (HMM) which has recently entered clinical trial. HMM is a synthetic *s*-triazine which showed broad-spectrum, if modest, activity in extensive clinical trials (Blum *et al.*, 1978; Legha *et al.*, 1976). It caused severe emesis and yet had to be given by mouth. Pentamethylmelamine (PMM) which is more water-soluble, was introduced in the hope that parenteral administration would alleviate the gastrointestinal toxicity. Unfortunately it proved to be even more emetic than HMM, indicating that this was a centrally mediated effect, and also caused severe sedation, even coma. These factors proved dose-limiting (Casper *et al.*, 1981; Goldberg *et al.*, 1980; Ihde *et al.*, 1981; Muindi *et al.*, 1983; Van Echo *et al.*, 1980). Anti-tumour activity in these Phase I trials was also disappointing.

HMM and PMM require metabolic activation in order to express significant cytotoxic activity *in vitro* (Rutty & Connors, 1977). The inhibitory effect of non-activated HMM and PMM can be reversed by removing the drug (Rutty *et al.*, 1983). However N-hydroxymethylmelamines, such as are formed during activation (Borkovec & DeMilo, 1967) are significantly more toxic *in vitro* (Rutty & Abel, 1980) and their effect is not reversed by drug removal (Rutty *et al.*, 1983).

Pharmacokinetic studies with PMM showed that metabolic activation occurred only very slowly in man compared with rodents and that this might account for its poor clinical activity (Rutty *et al.*, 1982). Trimelamol was therefore developed for clinical use since, as a melamine bearing N-hydroxymethyl groups, it does not require metabolic activation.

It had been noted that Trimelamol caused markedly less sedation than PMM in rodents (Newell *et al.*, 1983). The pharmacokinetics of Trimelamol and PMM in plasma and whole brain were therefore investigated in the mouse to see whether the brain levels achieved would correlate with the observed differences in neurotoxicity. Plasma protein binding and lipophilicity were examined in an attempt to explain these findings.

### Materials and methods

### *Chemicals*

PMM was synthesised by Prof. W.C.J. Ross at the Chester Beatty Research Institute. Trimelamol was synethesised by Warner–Lambert (Ann Arbor, Michigan, USA) for the NCI (NSC 283162).

Analytical grade reagents used in HPLC and preparation of buffers were supplied by May and

Correspondence: I.R. Judson.
Received 1 November 1985; and in revised form, 7 January 1986.

© The Macmillan Press Ltd., 1986

Baker Ltd. (Dagenham, Essex, UK), BDH Chemicals Ltd. (Poole, Dorset, UK), and Fisons Ltd. (Loughborough, Leics, UK). Cremophor EL(R) was obtained from Sigma Chemical Co. Ltd. (Poole, Dorset, UK).

*HPLC*

High performance liquid chromatography (HPLC) was performed using a Waters Associates Model ALC/GPC204 chromatograph (Waters Associates, Milford, Mass., USA) equipped with a model 480 variable wavelength detector. The method for analysing PMM (CB10-370), $N^2$-hydroxymethyl-$N^2$, $N^4$, $N^4$, $N^6$, $N^6$-pentamethylmelamine (HMPMM, CB10-369) and $N^2$, $N^4$-dihydroxymethyl-$N^2$, $N^4$, $N^6$, $N^6$-tetramethylmelamine (DHMtetraMM, CB10-419) utilised a 15 cm × 4.6 mm column containing Spherisorb 5 μm hexyl (C6) packing (Phase Sep Ltd., Queensferry, Clwyd, UK) protected by a 6.5 × 2.1 mm precolumn containing Co:PELL ODS packing (Whatman Ltd., Maidstone, Kent, UK). Isocratic elution was carried out with 50% methanol/50% ammonium formate at pH 6.3 running at 1.5 ml $min^{-1}$. Detection was by UV absorption at 225 nm, and peaks were quantified by electronic integration using a Trilab II data analysis system (Trivector Scientific Ltd., Sandy, Beds., UK). Twenty-five μl samples were injected automatically and the samples kept at 4°C. The separation was carried out at a constant temperature of 16°C.

The HPLC for Trimelamol used an octyl (C8) packing material and isocratic elution at 2.0 ml $min^{-1}$ with 30% methanol/70% 0.05 M ammonium bicarbonate pH 8.1. Detection was similarly by UV absorption at 225 nm.

Finally $N^2$,$N^4$,$N^6$-trimethylmelamine (TriMM) was analysed using a C8 column and isocratic elution at 2.0 ml $min^{-1}$ with 9% acetonitrile/91% 0.05 M ammonium bicarbonate. Detection was by UV absorbtion at 225 nm.

*Pharmacokinetics*

Male Balb $C^-$ mice, body weight 20–28 g were treated with PMM or Trimelamol at a dose of 90 mg $kg^{-1}$, the $ED_{90}$ versus the PC6 tumour (Rutty *et al.*, 1985). The drugs were dissolved in DMSO and diluted with 5% dextrose pH 7.4 to give a drug concentration of 4.5 mg $ml^{-1}$ and DMSO concentration of 5%. The drugs were administered intraperitoneally (i.p.), three mice per time point, at a constant volume of 0.2 ml per 10 g body weight. At regular intervals 5–60 min following injection, mice were anaesthetised with diethyl ether and blood withdrawn by direct cardiac puncture using pre-cooled syringes. The blood was placed in heparinised ice-cold tubes and centrifuged at 1800 *g* and 4°C for 10 min. The plasma was precipitated with 2 vol ice-cold methanol and again spun for 10 min at 4°C. The supernatant was removed and analysed by HPLC. After bleeding the animals were killed, the brains removed and placed in 10 ml ice-cold isotonic KCl solution (1.15%). The brains were washed in further KCl solution, blotted dry, weighed and hand-homogenised on ice in 4 vol (4 ml $g^{-1}$ wet tissue) 1.15% KCl. The suspension was precipitated with 2 vol ice-cold methanol, spun at 1800 *g* and 4°C for 10 min and this supernatant also analysed by HPLC. Standards were prepared in human plasma.

A single time point experiment was also performed to investigate the effect of a surfactant formulation on the CNS uptake of Trimelamol. The drug was given i.v. at 230 mg $kg^{-1}$, dissolved either in 5% dextrose pH 7.4 at 4.5 mg $ml^{-1}$ or in 10% Cremophor EL/5% dextrose pH 7.4 at the same concentration. Three mice were treated with each formulation and plasma and brain samples obtained 30 min after injection and prepared as above.

The exponential function $C = Ae^{-\beta t}$ (where $C$ is the drug plasma concentration, $A$ the constant, $\beta$ the first-order rate constant and $t$ the time after the end of drug administration) was fitted to the data by a non-linear least squares method in order to determine the half-lives (Dixon, 1980).

*Stability of Trimelamol*

The stability of Trimelamol at room temperature was measured in 5% dextrose pH 7.4 and in 10% Cremophor/dextrose pH 7.4. Serial estimations were performed by HPLC at intervals of 15–30 min over 5 h. The first order rate constants and half-lives were calculated as described above.

*Plasma protein binding*

The plasma protein binding of PMM, Trimelamol, HMPMM, DHtetraMM and TriMM was determined by ultrafiltration using the Amicon micropartition system (Amicon Corporation, Danvers, Mass., USA). The drugs were dissolved in water or DMSO and then diluted in human plasma or water to give a final drug concentration of 100 μg $ml^{-1}$ and varying DMSO concentrations of 5%, 0.1% or 0%. Each assay was performed in triplicate and consisted of incubation at 37°C for 3 min followed by centrifugation in ultrafiltration chambers containing YMB protein elimination filters at 2000 *g* and 15°C for 10 min. Owing to the chemical instability of N-hydroxymethylmelamines control samples were also prepared in plasma and treated identically apart from ultrafiltration in order to correct for chemical breakdown during the procedure. The water controls were performed in order to exclude a possible error due to binding of drug to the filters. The ultrafiltrates and controls were mixed with 2 vol ice-cold methanol and spun at 1800 *g* and 4°C for 10 min. The samples were analysed using the HPLC assays described above. Protein binding was calculated as plasma concentration minus ultrafiltrate concentration/plasma concentration × 100%.

### Results

The brain and plasma concentration profiles for PMM and Trimelamol following i.p. administration at 90 mg $kg^{-1}$ are shown in Figures 1 and 2, and summarised in Table I. The concentration of PMM in mouse brain was found to closely match the plasma concentration at all times and a mean value of 1.04 was obtained for the brain/plasma ratio. In contrast the Trimelamol brain concentration rose slowly over 30 min but never exceeded 1 μg $g^{-1}$.

**Figure 1** Plasma (—●—), and brain (--▲--), pharmacokinetics of PMM 90 mg $kg^{-1}$ i.p. in Balb $C^-$ mice.

**Figure 2** Plasma (●), and brain (▲), pharmacokinetics of Trimelamol 90 mg $kg^{-1}$ i.p. in Balb $C^-$ mice.

The values obtained may be overestimates, since a small amount of contamination would have occurred due to blood in the cerebral vessels. In addition such small concentrations were close to the limits of detection of the assay. The mean brain/plasma ratio over 60 min was <0.1.

The brain and plasma concentrations of Trimelamol following i.v. administration at 230 mg $kg^{-1}$ in 5% dextrose or 10% Cremophor/dextrose are summarised in Table II. Although a dose-related increase in plasma and brain concentration at 30 min was observed, the brain/plasma ratio was little elevated at 0.14 for the 5% dextrose formulation. Administration of the drug in 10% Cremophor resulted in profound acute sedation, and much higher concentration of Trimelamol in both brain and plasma were observed. The brain/plasma ratio at 30 min was unchanged at 0.15.

Trimelamol was quite stable in 5% dextrose at pH 7.4 and room temperature with an elimination half-life of 417.5 min (±6.3 s.e.). However the addition of 10% Cremophor stabilised the drug still

**Table I** Plasma and brain concentrations of PMM and Trimelamol in Balb $C^-$ mice following bolus injection at 90 mg kg$^{-1}$ i.p.

| *Drug* | *Peak plasma conc.* ($\mu g\, ml^{-1}$)[a] | *Plasma conc. at 30 min* ($\mu g\, ml^{-1}$) | *Plasma* $t_{1/2}\beta$ (*min*) | *Peak brain conc.* ($\mu g\, g^{-1}$) | *Brain conc. at 30 min* ($\mu g\, g^{-1}$) | *Mean brain/ plasma ratio* over 60 min |
|---|---|---|---|---|---|---|
| PMM | 18.0 (±1.0) (5 min) | 1.7 (±1.0) | 7.1 (±0.6) | 25.7 (±0.9) (5 min) | 1.25 (±0.12) | 1.04 (±0.2) (range 0.5–1.6) |
| Trimelamol | 33.8 (±6.0) (5 min) | 4.7 (±0.7) | 8.8 (±1.1) | 0.54 (±0.14) (30 min) | 0.54 (±0.14) | 0.008 (±0.07) (range 0.002–0.5) |

[a]Concentrations given are mean values from 3 animals (±s.e.).

**Table II** CNS penetration of Trimelamol 230 mg kg$^{-1}$ i.v. – Effect of 10% Cremophor EL

| *Formulation vehicle* | *Plasma conc. at 30 min* ($\mu g\, ml^{-1}$)[a] | *Brain conc. at 30 min* ($\mu g\, g^{-1}$) | *Brain/plasma* ratio |
|---|---|---|---|
| 5% Dextrose | 26.4 (±2.8) | 3.8 (±0.9) | 0.14 (±0.02) |
| 10% Cremophor/Dextrose | 70.3 (±5.8) | 10.8 (±1.0) | 0.15 (±0.003) |

[a]Concentrations given are mean values from 3 animals (± s.e.).

further giving a $t_{1/2}\beta$ of 730.6 min (±34.2 s.e.), i.e. an increase of 75%.

The results of the plasma protein binding studies are given in Table IIIa and show that Trimelamol is much less strongly protein bound than PMM, i.e. only 16.8% compared with 68.2%. It is apparent that protein binding is proportional to the number of methyl groups on the melamine ring. Repeat experiments in the presence of DMSO showed no significant effect on the protein-binding of PMM and no effect on that of Trimelamol, see Table IIIb. Neither drug bound to the protein elimination filters.

### Discussion

A marked difference was found in the ability of PMM and Trimelamol to penetrate the CNS of mice. PMM appeared to enter the brain rapidly with no detectable lag phase. Trimelamol, in contrast, entered the brain very poorly, its concentration never exceeding 1 $\mu g\, g^{-1}$. Precise conclusions about the kinetics of Trimelamol in the CNS are difficult to draw owing to the limits of accuracy of this experiment. However, it is clear that for PMM the brain/plasma ratio of ~1.0 indicates the lack of a 'blood–brain barrier' for this drug whereas Trimelamol is largely excluded from the CNS.

**Table IIIa** Plasma protein binding of melamines

| *Drug* | $R_1$ | $R_2$ | $R_3$ | *% Protein binding* |
|---|---|---|---|---|
| PMM | H | $CH_3$ | $CH_3$ | 68.2 |
| HMPMM | $CH_2OH$ | $CH_3$ | $CH_3$ | 63.0 |
| DHM-tetraMM | $CH_2OH$ | $CH_2OH$ | $CH_3$ | 36.2 |
| Trimelamol | $CH_2OH$ | $CH_2OH$ | $CH_2OH$ | 16.8 |
| TriMM | H | H | H | 13.0 |

**Table IIIb** Effect of DMSO on plasma protein binding

| *DMSO conc.* (%) | *Plasma protein binding* (%) Trimelamol | PMM |
|---|---|---|
| 0 | 16.8 | 68.2 |
| 0.1 | 14.6 | 71.2 |
| 5 | 15.3 | 62.1 |

Pentamethylmelamine (PMM)

$N^2,N^4,N^6$, Trihydroxymethyl-$N^2,N^4,N^6$, Trimethylmelamine (Trimelamol)

| | PMM | Trimelamol |
|---|---|---|
| Octanol/water partition | 67 | 2.5 |

**Figure 3** Structures and partition coefficients of PMM and Trimelamol.

Stewart *et al.* (1983) found high levels of PMM and its metabolites in human brain tumours, but only the metabolite, $N^2$-monomethylmelamine, was detectable in the adjacent normal brain. We also found higher levels of the decomposition products of both PMM and Trimelamol in mouse CNS compared with the parent compounds (unpublished data). However, as Stewart *et al.* indicated, the abnormal blood supply to cerebral tumour causes disruption of the blood–brain barrier. Therefore their findings are not directly comparable, and the dose of PMM used in their studies was very small.

This difference in CNS penetration between PMM and Trimelamol correlates very well with the consistently observed difference in neurotoxicity between the two drugs. PMM causes marked sedation in mice and rats and impairs the righting reflex whereas Trimelamol causes little or no sedation even at the maximum tolerated dose (Rutty *et al.*, 1985).

The effect of Cremophor EL on the CNS penetration of Trimelamol was investigated following the observation that its use in formulation was associated with an increase in neurotoxicity. Mice given Trimelamol in 10% Cremophor/5% dextrose became acutely sedated and sometimes fitted. On recovery the animals remained hypokinetic and rather ataxic for some hours. This was associated with an increase in early deaths but no change in the actual $LD_{50}$ (unpublished observations). The increase in neurotoxicity was confirmed in these experiments which were performed at $230 mg kg^{-1}$, a lethal dose of Trimelamol known to cause mild sedation. The increase in Trimelamol brain concentration in the presence of Cremophor was therefore expected. Other surfactants, e.g. polysorbate 80, are known to change the volume of distribution of certain drugs and may enhance their uptake into the CNS possibly by disrupting the blood–brain barrier (Azmin *et al.*, 1985). The reason for the increase in plasma concentration at 30 min is unclear. We found that the *in vitro* half-life of Trimelamol in 5% dextrose pH 7.4 was increased by 75% with the addition of 10% Cremophor. However, we have not confirmed that this also leads to an increase in the *in vivo* half-life. Whilst conferring some advantages in terms of solubility and stability the use of this particular surfactant was clearly associated with an unacceptable increase in Trimelamol neurotoxicity.

In conclusion the CNS penetration of Trimelamol is significantly less than that of PMM. This difference is clearly not due to a difference in plasma protein binding since PMM is much more highly protein bound, and the use of DMSO in the pharmacokinetic experiments would not have interfered with this. An explanation is more likely to be found in the marked differences in lipophilicity between the two compounds. Figure 3 shows the chemical structures and values for the octanol/water partition coefficients for PMM and Trimelamol as determined by Cumber & Ross (1977). The relatively low lipophilicity of Trimelamol seems likely to be responsible for its reduced CNS penetration and hence reduced neurotoxicity. In a Phase I trial at the Royal Marsden Hospital the drug has proved less emetic than PMM in man and causes little or no sedation, as well as showing encouraging signs of anti-tumour activity.

### References

AZMIN, M.N., STUART, J.F.B. & FLORENCE, A.T. (1985). The distribution and elimination of methotrexate in mouse blood and brain after concurrent administration of polysorbate 80. *Cancer Chemother. Pharmacol.*, **14**, 238.

BLUM, R.H., LIVINGSTON, R.B. & CARTER, S.K. (1978). Hexamethylmelamine – A new drug with activity in solid tumours. *Eur. J. Cancer*, **9**, 195.

BORKOVEC, A.B. & DEMILO, A.B. (1967). Insect chemosterilants V. Derivatives of melamine. *J. Med. Chem.*, **10**, 457.

CASPER, E.S., GRALLA, R.J., GARRETT, R.L. & 5 others (1981). Phase I and pharmacological studies of pentamethylmelamine administered by 24 hour infusion. *Cancer Res.*, **42**, 1402.

CUMBER, A.J. & ROSS, W.C.J. (1977). Analogues of hexamethylmelamine. The anti-neoplastic activity of derivatives with enhanced water solubility. *Chem. Biol. Interactions*, **17**, 349.

DIXON, W.J. (ed). (1980). *BMD Biomedical Computer Programs, X-series supplement.* University of California Press: Berkeley, California. p. 177.

GOLDBERG, R.S. GRIFFIN, J.P., McSHERRY, J. W. & KRAKOFF, I.H. (1980). Phase I study of pentamethylmelamine. *Cancer Treat. Rep.*, **64**, 1319.

IHDE, D.C., DUTCHER, J.S., YOUNG, R.C. & 5 others (1981). Phase I trial of pentamethylmelamine: A clinical and pharmacologic study. *Cancer Treat. Rep.*, **65**, 755.

JUDSON, I.R., RUTTY, C.J., ABEL, G., GUMBRELL, L., HARRAP, K.R. & CALVERT, A.H. (1985). Phase I trial of $N^2,N^4,N^6$-trihydroxymethyl-$N^2,N^4,N^6$-trimethylmelamine (Trimelamol) a new anti-tumour *s*-triazine. *Proc. Am. Ass. Clin. Oncol.*, **4**, 31.

LEGHA, S.S., SLAVIK, N. & CARTER, S.K. (1976). Hexamethylmelamine – An evaluation of its role in the therapy of cancer. *Cancer*, **38**, 27.

MUINDI, J.R.F., NEWELL, D.R., SMITH, I.E. & HARRAP, K.R. (1983). Pentamethylmelamine (PMM): Phase I clinical and pharmacokinetic studies. *Br. J. Cancer*, **47**, 27.

NEWELL, D.R., RUTTY, C.J., MUINDI, J.R.F. & HARRAP, K.R. (1983). Experimental studies on trimethyltrimethylolmelamine as an alternative to hexamethylmelamine (HMM) and pentamethylmelamine (PMM). *Br. J. Cancer*, **14**, 281.

RUTTY, C.J. & ABEL, G. (1980). *In vitro* cytotoxicity of the methylmelamines. *Chem. Biol. Interactions*, **29**, 235.

RUTTY, C.J. & CONNORS, T.A. (1977). *In vitro* studies with hexamethylmelamine. *Biochem. Pharmacol.*, **26**, 2385.

RUTTY, C.J., JUDSON, I.R., ABEL, G. & 3 others (1986). Preclinical toxicology, pharmacokinetics and formulation of $N^2,N^4,N^6$-trihydroxymethyl-$N^2,N^4,N^6$-trimethylmelamine (Trimelamol) a water soluble cytotoxic *s*-triazine which does not require metabolic activation. *Cancer Chemother. Pharmacol.* (in press).

RUTTY, C.J., NEWELL, D.R., MUINDI, J.R.F. & HARRAP, K.R. (1982). The comparative pharmacokinetics of pentamethylmelamine in man, rat and mouse. *Cancer Chemother. Pharmacol.*, **8**, 105.

RUTTY, C.J., NEWELL, D.R., MUINDI, J.R.F. & HARRAP, K.R. (1983). Development of potential clinical alternatives to hexamethylmelamine. In *The Control of Tumour Growth and Its Biological Bases*, Davis, W. *et al.* (eds) p. 180. Academie Verlag: Berlin.

STEWART, D.J., BENVENÜTO, J.A., LEAVENS, M. & 4 others (1983). Human central nervous system pharmacology of pentamethylmelamine and its metabolites. *J. Neuro. Oncol.*, **1**, 357.

VAN ECHO, D.A., CHIUTEN, D.F., WHITACRE, M. & 3 others (1980). *Cancer Treat. Rep.*, **64**, 1335.